# Mapping the Epitopes of a Neutralizing Antibody Fragment Directed against the Lethal Factor of *Bacillus anthracis* and Cross-Reacting with the Homologous Edema Factor

**DOI:** 10.1371/journal.pone.0065855

**Published:** 2013-05-31

**Authors:** Philippe Thullier, Arnaud Avril, Jacques Mathieu, Christian K. Behrens, Jean-Luc Pellequer, Thibaut Pelat

**Affiliations:** 1 Unité de Biotechnologie des Anticorps, et des Toxines, Institut de Recherche Biomédicale des Armées, La Tronche, France; 2 Département de Microbiologie, Institut de Recherche Biomédicale des Armées, La Tronche, France; 3 Laboratoire Français du Fractionnement et des Biotechnologies, Les Ulis, France; 4 Service de Biochimie et Toxicologie Nucléaire, CEA, IBEB, Bagnols-sur-Cèze, France; Wadsworth Center, New York State Dept. Health, United States of America

## Abstract

The lethal toxin (LT) of *Bacillus anthracis,* composed of the protective antigen (PA) and the lethal factor (LF), plays an essential role in anthrax pathogenesis. PA also interacts with the edema factor (EF, 20% identity with LF) to form the edema toxin (ET), which has a lesser role in anthrax pathogenesis. The first recombinant antibody fragment directed against LF was scFv 2LF; it neutralizes LT by blocking the interaction between PA and LF. Here, we report that scFv 2LF cross-reacts with EF and cross-neutralizes ET, and we present an *in silico* method taking advantage of this cross-reactivity to map the epitope of scFv 2LF on both LF and EF. This method identified five epitope candidates on LF, constituted of a total of 32 residues, which were tested experimentally by mutating the residues to alanine. This combined approach precisely identified the epitope of scFv 2LF on LF as five residues (H229, R230, Q234, L235 and Y236), of which three were missed by the consensus epitope candidate identified by pre-existing *in silico* methods. The homolog of this epitope on EF (H253, R254, E258, L259 and Y260) was experimentally confirmed to constitute the epitope of scFv 2LF on EF. Other inhibitors, including synthetic molecules, could be used to target these epitopes for therapeutic purposes. The *in silico* method presented here may be of more general interest.

## Introduction

In 2001, the intentional release of anthrax spores through the U.S. postal system confirmed that *Bacillus anthracis* can cause high morbidity and mortality, despite the use of powerful antibiotherapy and resuscitation techniques. The pathogenesis of *B. anthracis* is largely due to a tripartite protein complex consisting of a component binding cellular receptors, the protective antigen (PA), and two catalytic components, the lethal factor (LF) and the edema factor (EF). PA and LF combine to form the lethal toxin (LT), and PA and EF combine to form the edema toxin (ET). However, only LT is recognized as being essential for anthrax pathogenesis (for a review, see [1]–[2]). EF and LF bind to PA with high affinities (K_D_  =  1 nM) [3]; their binding is competitive and involves their N-terminal domains, which present a conserved structure [4]–[6].

For antibiotic treatments of anthrax to be effective, they must be administered rapidly after infection [7] as lethal amounts of anthrax toxins are quickly secreted into the bloodstream. Antibiotic efficacy is also limited by the existence of *B. anthracis* antibioresistance [8]–[10]. However, it was demonstrated in animal models of anthrax that the passive transfer of neutralizing antibodies directed against either PA or LF can improve the outcome of the disease [11]. Consequently, considerable efforts have been devoted, since 2001, to the development of recombinant antibodies to be used to complement antibiotic therapy (for a review, see [12]–[13]), and they resulted in the recent FDA approval of raxibacumab for the treatment of inhalational anthrax [14]. However, concerns have been raised about the use of anti-PA antibodies alone [15], because it was feared that PA could be naturally or voluntarily modified so as to escape binding by anti-PA antibodies while retaining its biological activity [16]. Consequently anti-LF antibodies have also been considered for anthrax therapy [15]. Another possible advantage of such antibodies is that they could potentially synergize with anti-PA antibodies [17]–[20].

The first recombinant anti-LF antibody fragment, scFv 2LF, was isolated using an original strategy, based on the construction of phage-displayed libraries from immunized macaques (*Macaca fascicularis*). Macaque and human antibodies are similar and, if needed for therapeutic purposes, the immunogenicity of macaque antibodies can be decreased by germline humanization [21]–[22]. The neutralization of the lethal toxin by scFv 2LF is due to the inhibition of the interaction between PA and LF, i.e. by the inhibition of the formation of the toxin [18]. This mechanism is shared by other LF-neutralizing antibodies [19]–[20], [23]–[25] but their epitopes remain to be accurately mapped.

One LF-neutralizing antibody, 10G4, cross-reacts with EF [23] and we hypothesized that scFv 2LF also cross-reacts with EF. This cross-reactivity was observed and it prompted us to map accurately the epitopes of scFv 2LF on both LF and EF, in a study involving four different steps. First, the cross-reactivity of scFv 2LF with EF was tested and the cross-neutralization of ET was characterized. Second, an *in silico* method was developed to identify regions exposed to the solvent and shared between LF and EF, as these regions were regarded as epitope candidates. In the third part, these epitope candidates were tested by mutating their residues to alanine, thereby mapping the epitope precisely. Lastly, the homolog of this epitope on EF was experimentally demonstrated to constitute the epitope of scFv 2LF on EF.

In this work, antigen residues were considered to be part of the epitope only if they contributed directly to antibody binding. Epitopes are generally composed of only a few such residues [26] and they can be identified by mutation to alanine [27]. This approach is based on the fact that interactions between antibodies and antigens depend on interactions between amino-acid side chains. The side chain of alanine is constituted of a methyl group thus it is very small, and substituting one of the key residues constituting an epitope with alanine weakens the interaction between the antigen and the antibody [28]. Therefore, the involvement of a residue in an epitope may be tested by mutating it to alanine: a mutation weakening the affinity for the antibody shows that the residue is part of the epitope. For epitope mapping generally, the first step is for whole regions regarded as epitope candidates to be mutated to alanine (or “shaved to alanine”). In a second step, the residues constituting the regions previously tested positively are each individually mutated to alanine (or “scanned to alanine”) to confirm and map precisely the epitope (for a review see [29]).

## Results

### ScFv 2LF cross-reacts with EF and cross-neutralizes ET

**Figure 1:**
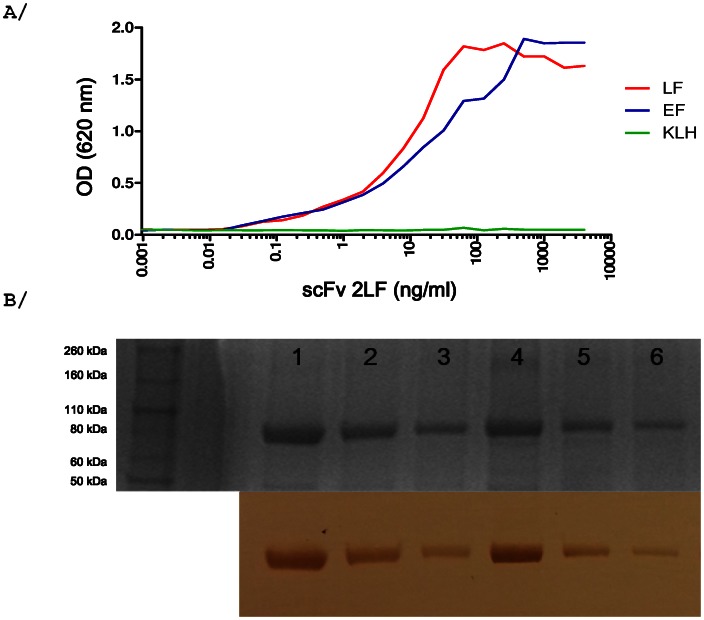
Evaluation of the reactivity of scFv 2LF with EF by ELISA and western blot. A/ Reactivity of scFv 2LF with EF in ELISA. Reactivity of scFv 2LF with EF is represented in blue, and LF (red) and KLH (green) were used as positive and negative controls, respectively. ScFv 2LF reacted with EF, though less strongly than with LF at high scFv concentrations. B/ Reactivity of scFv 2LF with EF in western blots under reducing conditions (lower part). EF (5, 2 and 1 µg/ml in lanes 4, 5 and 6, respectively) and LF as a positive control (5, 2 and 1 µg/ml in lanes 1, 2 and 3, respectively) were separated by SDS-PAGE electrophoresis (upper part) and their sizes verified to be 90 kDa by separation under reducing conditions. ScFv 2LF reacted similarly with EF and LF under reducing conditions.

**Figure 2:**
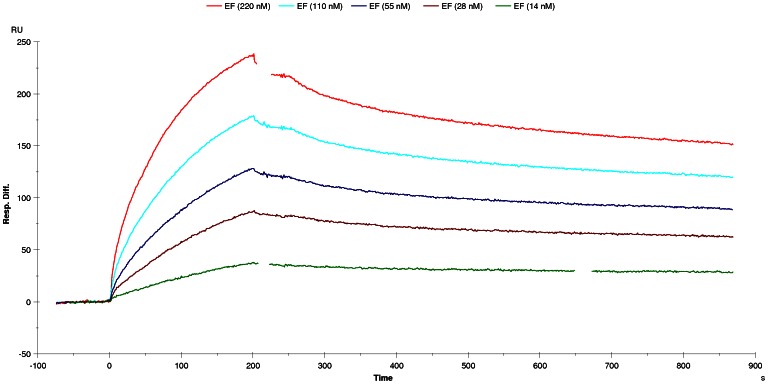
Biacore experiment to measure the affinity of scFv 2LF for EF. Sensorgrams were obtained at EF concentrations of 220 nM (red curve), 110 nM (light blue), 55 nM (dark blue), 28 nM (brown) and 14 nM (green) on a CM5 chip sensitized by 250 response units of scFv 2LF. The affinity of scFv 2LF for EF was measured as 5 nM.

In ELISA, and in western blot under reducing conditions, scFv 2LF reacted with both EF and LF (figure 1). The reactivity under reducing conditions indicated that the scFv 2LF epitopes on LF and EF are essentially linear. In a Biacore experiment, the affinity of scFv 2LF for EF was found to be 5 nM (figure 2), which is 5-fold lower than the affinity of scFv 2LF for LF (1.02 nM) [18]. This difference indicates that the epitopes of scFv 2LF on EF and LF are not strictly identical.

**Figure 3:**
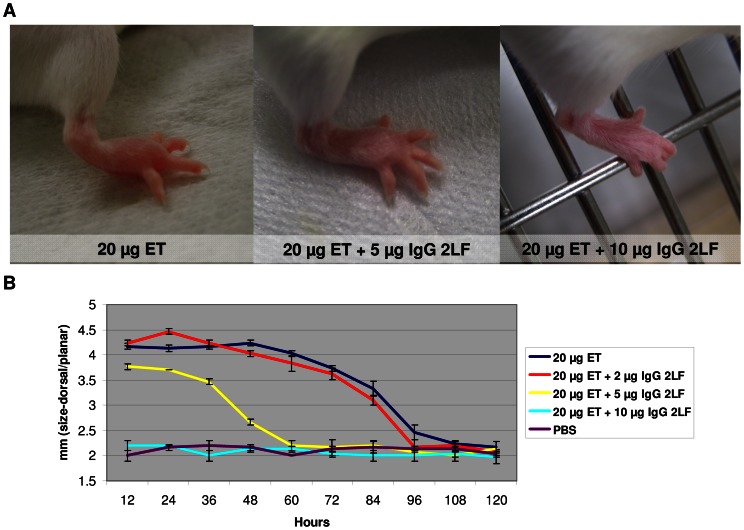
IgG 2LF inhibits the formation of edema induced by the edema toxin (ET). (A) Photographs taken 12 hours after injection in mouse footpads of 20 µg ET (left picture), or of 20 µg ET pre-mixed with 5 µg (middle picture) or with 10 µg (right picture) of IgG 2LF. Ten µg of IgG 2LF premixed with 20 µg ET completely inhibited edema formation, whereas 5 µg of IgG 2LF reduced both the size and the duration of the edema; 2 µg of IgG 2LF had no appreciable effect. (B) Evolution of the sizes (dorsal/plantar) of mouse footpads after injection of 20 µg ET (blue), or of 20 µg ET pre-mixed with 2 µg (red) or 5 µg (yellow) or 10 µg (light blue) of IgG 2LF. Injection of PBS only (purple) was used as a negative control. Curves represent triplicate measurements for each timepoint. Pre-mixing with 10 µg of IgG 2LF completely abolished the formation of edema by 20 µg ET, and pre-mixing with 5 µg of IgG 2LF limited the size of the edema and reduced its duration. Pre-mixing of 20 µg ET with 2 µg of IgG 2LF had no appreciable effect.

The capacity of scFv 2LF to neutralize ET was tested *in vivo*. Twentyµg of edema toxin (ET) injected into mouse footpads caused edema (figure 3A, left panel), culminating at the 12^th^ hour after injection and returning to normal after 108 hours (figure 3B). The edema was not reduced by premixing 2 µg of IgG-2LF with 20 µg ET. However, premixing 5 µg of IgG 2LF with 20 µg ET resulted in the edema being 25% smaller than that caused by ET alone, and the duration of edema (60 h vs. 108 h) being 45% shorter (figure 3A, middle panel, and figure 3B). Tenµg of IgG 2LF premixed with 20 µg ET completely inhibited edema formation (figure 3A, right panel, and figure 3B), and injection of PBS only did not induce edema. These data demonstrate that, in addition to neutralizing LT, scFv 2LF also neutralizes ET.

### Identification of epitope candidates by a new *in silico* method

**Figure 4:**
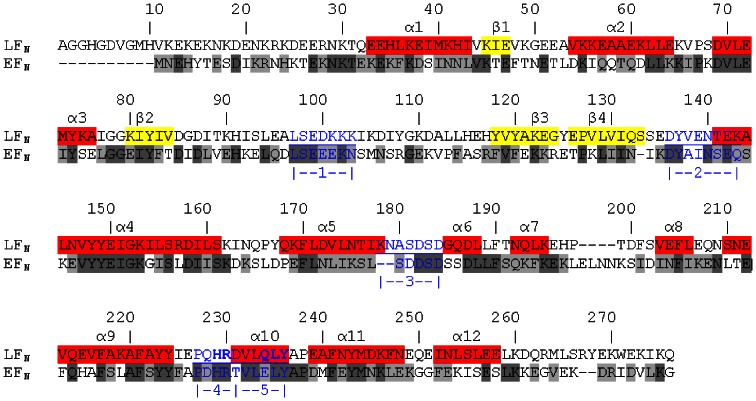
Alignment of the sequences of LF_N_ and EF_N_, and prediction of their secondary structures according to the LF 3D-structure (1J7N). The α helixes (1 to 12) are colored in red and β strands (1 to 4) in yellow. The residues that are identical, very similar and different between LF_N_ and EF_N_ are highlighted in the EF_N_ sequence in dark gray, light gray and white, respectively. The five linear regions identified as epitope candidates are indicated in blue, underlined and numbered: region 1 is LF(97–103), region 2 is LF(136–143), region 3 is LF(178–184), region 4 is LF(227–231) and region 5 is LF(232–236). The residues of LF_N_ constituting the scFv 2LF epitope are indicated in bold.

ScFv 2LF blocks the interaction between LF and PA so that its epitope is presumably located in the N-terminal domain of LF, LF_N_ [18]. Also, scFv 2LF cross-reacts with EF and LF, so its epitope is presumably highly conserved between the two proteins. To interact with an antibody, an epitope has indeed to be exposed to the solvent. Regions highly conserved between LF_N_ and its homolog, the N-terminal domain of EF (EF_N_), were thus considered to be epitope candidates if they were exposed to the solvent. An *in silico* method was designed to search for such regions, and its main features were that (i) it was based on a sliding window of five residues, corresponding to the lengths of most epitopes, and residues constituting the extremities of candidate sequences were analyzed in detail (ii) the mean identity between the LF_N_ and EF_N_ sequences was calculated as equal to 35% (figure 4), and this value was used as a threshold, above which regions were selected as candidates if they appeared to be exposed to the solvent (iii) the solvent exposure of candidates was verified statistically.

**Figure 5:**
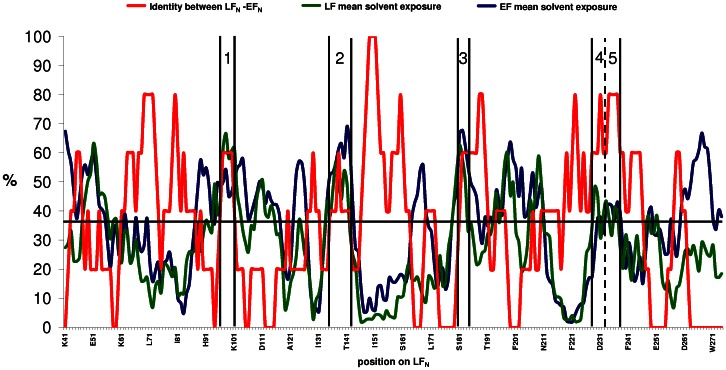
Identification of epitope candidates by the combined analysis of sequence identity and solvent exposure. The curve in red represents the percentage of identity between LF_N_ and EF_N_, averaged over a sliding window of five residues. The mean identity between LF_N_ and EF_N_, 35% (horizontal black line), was chosen as the threshold above which regions were selected as epitope candidates. The curves in green and blue indicate the solvent exposure of LF_N_ and EF_N_, respectively, averaged over a sliding window of five residues. The five regions of LF_N_ and EF_N_ exposed to the solvent, and sharing an identity higher than the threshold, were selected as epitope candidates. These regions are numbered 1 to 5, and their ends are indicated by vertical black lines.

Starting from the N-terminus, the first region exposed to the solvent and conserved between LF_N_ and EF_N_ is located between positions 96 and 102 (figure 5), as it presents 56% identity. Residue 96 is an alanine, thus it is unlikely to participate significantly in the scFv 2LF binding and it was excluded from the candidate region. Position 103 was added as the corresponding residues in LF (K103) and EF (N103) are exposed to the solvent. The first epitope candidate was thus refined as LF(97–103) and its mean solvent exposure was 53% (table 1).

**Table 1:** Mean identity between each epitope candidate in LF and the homologous region in EF, and mean solvent exposure of each epitope candidate.

Epitope candidate	length	Mean identity between the amino-acid sequence and the homologous region in EF (%)	Mean solvent exposure (%)
LF(97–103)	7	57	53
LF(136–143)	8	50	47
LF(178–184)	7	43	52
LF(227–231)	5	60	48
LF(231–236)	6	67	44

The identities between EF and LF epitope candidates are higher than the threshold, equal to 35%. The calculated mean solvent exposure for the five epitope candidates is 48%.

The second selected candidate region is located between residues 136 and 142, and its percentage identity is 57% (figure 5). Residue K143 was added to this candidate region as it is exposed on LF. The second candidate region retained for further testing was thus LF(136–143), with a mean solvent exposure of 47% (table 1).

The region between LF residues 180 and 184 shares 60% identity with EF, and is exposed to the solvent on both proteins (figure 5). It was refined as LF(178–184), as residues K178 and N179 were added to compensate for the lack of homologs on EF. The mean solvent exposure of LF(178–184) is 52% (table 1).

The region located between 227 and 237 is very similar in LF_N_ and EF_N_ (73%), and exposed to the solvent (figure 5). Because residue 237 is an alanine, only the region between residues 227 and 236 was retained for further study. It was divided into two regions, LF(227–231) and LF(231–236), to limit the number of residues needing to be tested in the following steps of the study. The mean solvent exposures of LF(227–231) and of LF(231–236) are 48% and 44%, respectively (table 1).

Five regions, LF(97–103), LF(136–143), LF(178–184), LF(227–231) and LF(231–236), were thus selected as epitope candidates and their mean solvent exposure is 48.1%. The rest of the LF_N_ and EF_N_ regions sharing an identity greater than 35% had a mean solvent exposure of 19.7%, which is significantly lower (p<0.0001) than the selected regions. The five selected LF regions were thus confirmed as epitope candidates ; they are constituted of 32 residues, thus far less than the 261 residues constituting LF_N_.

### Evaluation of the epitope candidates by alanine shaving

If the mutation to alanine (or “shaving to alanine”) of a region selected as an epitope candidate reduced the binding of the mutated LF to scFv 2LF, compared to the unmutated LF, the region was selected for subsequent analysis.

**Figure 6:**
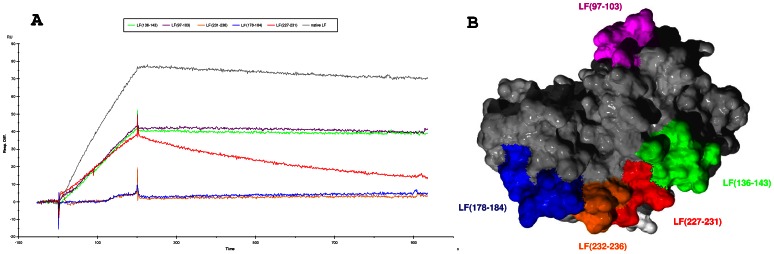
Biacore experiment to evaluate the reactivity of the five LF variants in which each epitope candidate had been shaved to alanine, and localization of these epitope candidates on a view of the LF_N_ surface. A/ The Biacore experiment was performed on a CM5 chip sensitized by scFv 2LF. The curve corresponding to the LF variant in which LF(97–103) was shaved is in purple, the curves corresponding to the shaving of LF(136–143), LF(178–184), LF(227–231) and LF(231–236) are in green, blue, red and orange, respectively. The positive control (commercially available LF) curve is in gray. Only the reactivities of LF variants in which LF(178–184), LF(227–231), LF(231–236) had been shaved were significantly (three-fold or more) lower than the positive control value. B/ The same color code as in A indicated the five epitope candidates on an apical view of the LF_N_ surface. The three regions selected after alanine shaving, LF(178–184), LF(227–231) and LF(231–236), are proximal. The figure was drawn using Swiss PDB viewer and Pov-ray rendering [49].

**Figure 7:**
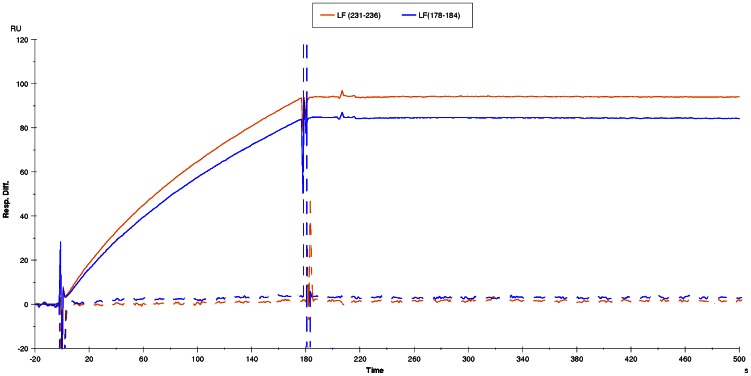
Biacore experiment to evaluate the reactivity between anti-LF polyclonal antibodies and the two LF variants, in which LF(178–184) or LF (231–236) had been shaved in alanine. A CM5 chip was sensitized using LF variants in which LF(178–184) (blue) or LF (231–236) (red) had been shaved in alanine, as these variants did not react with scFv 2LF and their presence and general conformation had to be verified. The diluted (1:100) serum of a macaque, drawn before immunization with LF,was used as a negative control and did not react with these two LF variants (dashed curves). The serum from the same animal after hyper-immunization with LF reacted with these two LF variants (continuous curves), showing that they had been immobilized on the CM5 chip. The curves are representative of two separate Biacore experiments, realized in the same conditions except for the variants used for sensitization.

First, it was verified that the unmutated LF produced in our laboratory had a reactivity for scFv 2LF (k_d_ =  2.3×10^−4^ s^−1^) equivalent to that of a commercially available LF (k_d_ =  2.1×10^−4^ s^−1^). The residues of region LF(97–103) were substituted with alanine and the interaction between the mutated LF and scFv 2LF was tested. The observed k_d_ was 1.32×10^−4^ s^−1^ (figure 6A), thus equivalent to the value observed for the unmutated LF, so that this region was not studied further. Similarly, mutating the residues of region LF(136–143) to alanine led to a k_d_ of 3×10^−4^ s^−1^ (figure 6A), and therefore this second epitope candidate was also rejected. Substitution of the residues of LF(178–184) and of LF( 231–236) caused a complete loss of reactivity with scFv 2LF. It was verified that these two variants still reacted with the anti-LF polyclonal antibodies (figure 7), showing that they had been immobilized on the sensorchip and that the loss of reactivity with scFv 2LF was not an artefact. These two regions were thus selected as candidates. Mutation of LF(227–231) to alanine reduced the reactivity with scFv 2LF to a k_d_ of 1.26×10^−3^ s^−1^; this result suggested that LF(227–231) contributes to the interaction with scFv 2LF but that it is not essential for this interaction, and it was included in subsequent evaluations. Thus, alanine shaving identified three regions, LF(178–184), LF(227–231) and LF(231–236), constituted of 17 residues, as candidates to be further studied by alanine scanning. These three regions are close to each other on the three-dimensional structure of LF (figure 6B).

### Precise identification of the epitope on LF and EF

**Figure 8:**
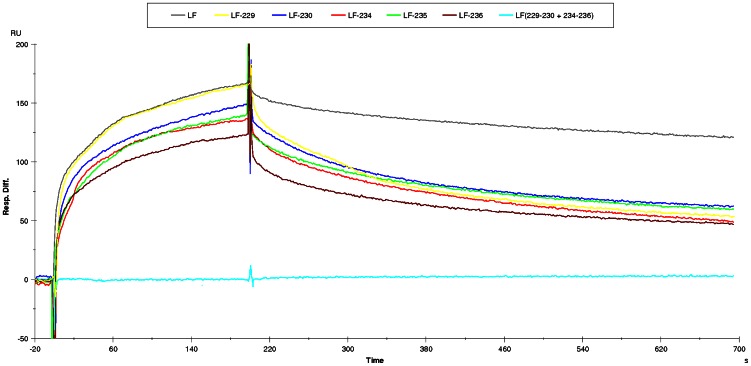
Biacore experiment to evaluate the reactivity between scFv 2LF and the five LF variants, in which H229, R230, Q234, L235 or Y236 had been individually mutated in alanine, or the LF variant where these five residues had been simultaneously mutated in alanine. A CM5 chip was sensitized with scFv 2LF. LF (control, in grey) and its five variants in which H229 (yellow), R230 (blue), Q234 (red), L235 (green) or Y236 (brown) had been individually mutated in alanine reacted in flux. The LF variant in which these five positions had been simultaneously mutated in alanine is presented in blue. Dilutions of LF and its variants giving an equivalent maximal signal are presented, to allow the comparison of the dissociation phases (after the maximal signal and the artefactual spikes) when dissociation constants are measured (between the 500^ th^ and 700^th^ seconds). Values were calculated on several curves for each variant, and are presented on table 2: the five variants with punctual mutations present at least a three-fold more rapid dissociation than the control, and the variant with the five simultaneous mutations presents no reactivity.

**Figure 9:**
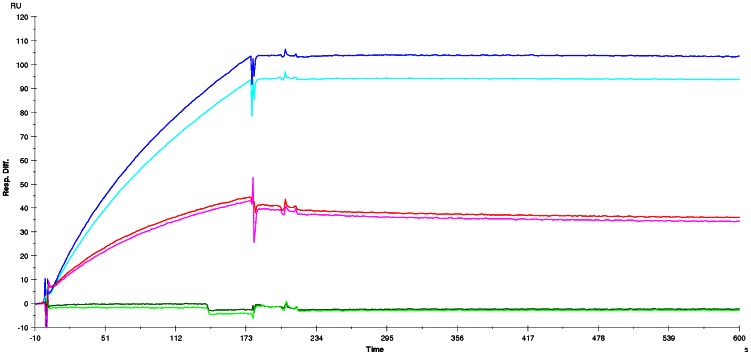
Biacore experiment to evaluate the reactivity between anti-LF polyclonal antibodies and LF, EF or their variants in which the epitope of scFv 2LF had been shaved in alanine. A CM5 chip was sensitized with LF (deep blue) or its variant (light blue) in which residues 229, 230, 234, 235 and 236 (corresponding to scFv 2LF epitope on LF) had been simultaneously substituted by alanine. Another CM5 chip was sensitized with EF (red) or its variant (rose) in which residues 253, 254, 258, 259 and 260 (corresponding to scFv epitope on EF) had been simultaneously mutated in alanine. The serum of a macaque drawn before or after immunization with LF was utilized in flux. The serum drawn before immunization did not react with LF nor with EF as expected (in green). The serum drawn after immunization reacted with both variants, though slightly less than with the parental molecules, showing that they had been immobilized on the chip and that their general conformations had been retained despite the shaving.

Each of the residues constituting LF(178–184), LF(227–231) and LF(231–236) were individually mutated to (“scanned to”) alanine. The mutations of residues H229, R230, Q234, L235 and Y236 weakened the binding between LF and scFv 2LF, as the corresponding values of k_d_ were at least 3-fold lower than that of the unmutated sequence (figure 8 and table 2). Mutation to alanine of the other residues constituting these three regions did not substantially modify LF reactivity. The LF variant in which H229, R230, Q234, L235 and Y236 were all replaced with alanine did not bind scFv 2LF, but it was verified that it still reacted with the anti-LF polyclonal antibodies (figure 9), demonstrating that the scFv 2LF epitope on LF is constituted by LF(229–230) plus LF(234–236). This epitope is linear as expected, but discontinuous.

**Table 2:** ScFv 2LF dissociation rate constants with each LF variant, measured by Biacore experiments.

	residues	k_d_ (s^−1^)
Shaving(SH)	97–103	1.32×10^−4^
	136–143	3×10^−4^
	178–184	/
	227–231	1.26×10^−3^
	231–236	/
Scanning	K178	2×10^−4^
	N179	2.12×10^−4^
	S181	3.1×10^−4^
	D182	3.9×10^−4^
	S183	2.9×10^−4^
	D184	1.3×10^−4^
	P227	5.6×10^−4^
	Q228	6×10^−4^
	H229	10×10^−4^
	R230	9.34×10^−4^
	D231	5.3×10^−4^
	V232	5.24×10^−4^
	L233	4.8×10^−4^
	Q234	9.54×10^-4^
	L235	8.95×10^−4^
	Y236	8.55×10^−4^
Native LF	/	2.3×10^−4^
Native EF	/	5.1×10^−4^

The five residues whose mutation to alanine resulted in a 3-fold, or larger, decrease of the dissociation constant are shown in bold.

We tested whether the homolog of this epitope in EF constitutes the epitope of scFv 2LF on EF. For this, a recombinant EF was produced in our laboratory and confirmed to present an reactivity with scFv 2LF similar to that of the commercially available EF (k_d_ = 5.1×10^−4^ s^−1^, table 2). The homologs of LF(229–230) and of LF(234–236) were identified by sequence alignment (figure 4) as EF(253–254) and EF(258–260), respectively. These five residues were all mutated to alanine and the mutated EF was not recognized by scFv 2LF but it reacted with the anti-LF polyclonal antibodies, showing that it retained its general conformation (figure 9). The epitope of scFv 2LF on EF is thus EF(253–254) plus EF(258–260).

### Identification of the epitope candidates by existing in *silico* methods

**Figure 10:**
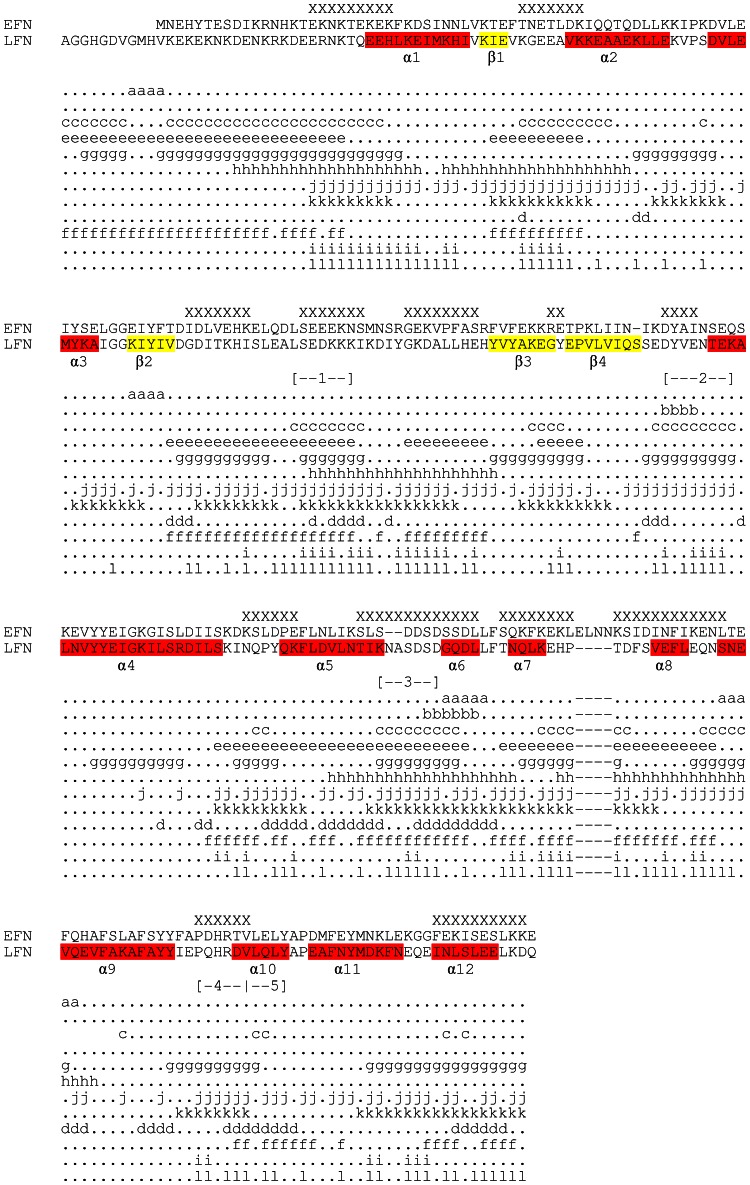
Results with web-based or stand-alone programs predicting 2LF epitopes. Results of 12 prediction methods used to predict 2LF epitopes are given as letters indicating the predictions and the method (a: BEPITOPE (TURNEE); b: BEPITOPE (TURN33); c: BepiPred; e: Ellipro; g: LEP-LP; h: BCPREDS; j: Epitopia; k: COBEpro; d: CBTOPE; f: Ellipro; i: DiscoTope; l: BEpro). The residues predicted as being part of the epitope are indicated by an “X” above the LF_N_ and EF_N_ alignment. The five epitope candidates identified by the *in silico* method developed in the present study are indicated with hyphens and numbered.

Twelve *in silico* prediction methods, available through web servers or stand-alone programs, were used to map on LF_N_ the epitope of scFv 2LF (figure 10). To be in a consensus epitope, a residue must have been predicted as part of the epitope by at least six of the 12 methods. Twelve consensus epitopes were identified: LF(27–35), LF(49–55), LF(86–91), LF(98–104), LF(109–116), LF(124–125), LF(136–139), LF(164–169), LF(176–188), LF(191–210), LF(227–232) and LF(252–261), including a total of 98 residues. Although this consensus region was large, it did not include three (LF(234–236)) of the five residues demonstrated in the present study as constituting the epitope.

## Discussion

The scFv 2LF was isolated from a phage-displayed library built from a macaque immunized with the LF sub-unit of the lethal anthrax toxin (LT). It binds LF and neutralizes LT by blocking the interaction between its two components, LF and PA [18]. Here, we show that scFv 2LF also binds EF (20% identity with LF), a component of the edema toxin (ET), constituted of EF and PA. An IgG was derived from scFv 2LF and was shown to neutralize ET *in vivo* (figure 3). The scFv 2LF was previously showed as competing with PA for the interaction with LF, and it was hypothesized that it also competes with PA for the interaction with EF. Because EF and LF interact with PA through their amino-terminal domains, LF_N_ and EF_N_, it was also hypothesized that the epitopes of scFv 2LF are located in these domains [4]–[6]. These epitopes were expected to be well conserved but not identical, because the K_D_ characterizing the interaction between scFv 2LF and EF is about 5-fold less favorable than the K_D_ characterizing the interaction between scFv 2LF and LF.

An *in silico* analysis was used to search for epitope candidates, defined as LF_N_ and EF_N_ regions exposed to the solvent and presenting a high degree of identity. The degree of identity was averaged over a sliding window of five residues, a common size for an epitope, and regions with identity greater than the mean identity between LF_N_ and EF_N_ were selected. Five epitope candidates totaling 32 amino acids were thereby identified in the candidate domain, LF_N_, composed of 261 amino acids. This elimination of 87% of the residues greatly facilitated the subsequent experimental investigations, based on mutations to alanine.

The performance of the *in silico* method developed here was compared with that of 12 existing methods. The consensus epitope candidates (the epitope candidates indicated by at least half of these methods) consisted of 98 amino acids, thus was three times larger than the epitope candidates identified by our analysis. Four out of five epitope candidates (LF(97–103), LF(136–143), LF(178–184), and LF(227–231)) identified in the present *in silico* study overlap with these consensus epitope candidates. Regarding the fifth epitope candidate identified by our analysis, LF(231–236), only residues 231 and 232 were among the consensus epitope candidates. Therefore, the consensus approach missed LF(234–236) though it constitutes three of the five residues of the epitope. Only four of the twelve pre-existing methods indicated LF(234–236) as epitope candidate ; these comparisons highlight the usefulness of the *in silico* analysis presented here.

Alanine shaving [30] and alanine scanning [27] are complementary approaches to determine experimentally which regions are involved in protein-protein interactions [31]–[32]: alanine shaving identifies regions containing an epitope, whereas alanine scanning maps precisely the epitope. Here, alanine shaving was utilized to test the five epitope candidates selected by the *in silico* study. Shaving LF(178–184) and LF(231–236) completely abolished the interaction with scFv 2LF, and shaving LF(227–231) partially reduced the interaction. In these three regions, alanine scanning identified five residues involved in the interaction with scFv 2LF: H229, R230, Q234, L235 and Y236. The simultaneous mutation of these five amino acids to alanine completely abolished LF reactivity with scFv 2LF, demonstrating that they constitute the epitope of scFv 2LF on LF. This epitope partially corresponds to the α helix 10, localized in the LF(231–236) region. Alanine scanning showed that LF(178–184) has no direct role in the interaction between LF and scFv 2LF, even though this region was tested positively by alanine shaving. It is possible that LF(178–184) contributes to the conformation of α helix 10, so that its role in scFv 2LF binding is indirect. This discordance between results obtained by alanine shaving and scanning exemplify that alanine scanning is not only useful for precise epitope mapping, but also necessary to confirm the results of alanine shaving.

Three of the amino acids constituting the scFv 2LF epitope, Q234, L235 and Y236, are included in the LF(231–236) region, and alanine shaving of this region completely abolished the interaction with scFv 2LF. These three residues are therefore essential for the interaction. The two other residues, H229 and R230, are located within the LF(227–231) region, whose shaving only reduced the strength of the interaction, so that their role is likely to be less important.

The homolog on EF of the scFv 2LF epitope in LF was localized by sequence alignment as EF(253–254) and EF(258–260). The mutation to alanine of these two regions confirmed that they constitute the scFv 2LF epitope of EF. The residues H229, R230, L235 and Y236, part of the scFv 2LF epitope on LF, are strictly conserved in EF, respectively at positions 253, 254, 259 and 260. The glutamine residue at position 234 in LF corresponds to a glutamic acid residue at position 258 in EF; physicochemical properties of these two amino-acids differ only slightly but these differences presumably explain the 5-fold weaker interaction of scFv 2LF with EF than with LF.

If no epitope candidate had been tested positively by mutations to alanine, a second series of candidates would have been defined. For this, the mean exposure of residues constituting LF_N_ (29%) would have been utilized as the solvent exposure threshold, in addition to the similarity threshold. These two thresholds, each defined as a mean value characterizing the antigens, would have identified five additional regions as epitope candidates, constituted of a total of 25 residues, and these regions would have been tested similarly than the first series.

Our first attempt to map the scFv 2LF epitope utilized five synthetic peptides corresponding to the candidates selected by the *in silico* analysis. Their reactivity with 2LF was tested by ELISA. The peptide corresponding to LF(97–103) was the only peptide that bound to the 2LF antibody, but it did not compete with LF for scFv 2LF binding (data not shown). For this reason, we used a different approach involving mutagenesis techniques guided by the same *in silico* results. As reported above, the mutagenesis experiments gave different results than the identification of LF(97–103) as epitope. This example illustrates how soluble peptides may present conformations different than those existing in the context of whole proteins, and consequently be misleading for epitope mapping (also see [20] for a discussion of peptide-antibody interactions). It also indicates that epitope mapping with peptides should be confirmed by competition experiments, which is not always the case in published studies.

**Figure 11:**
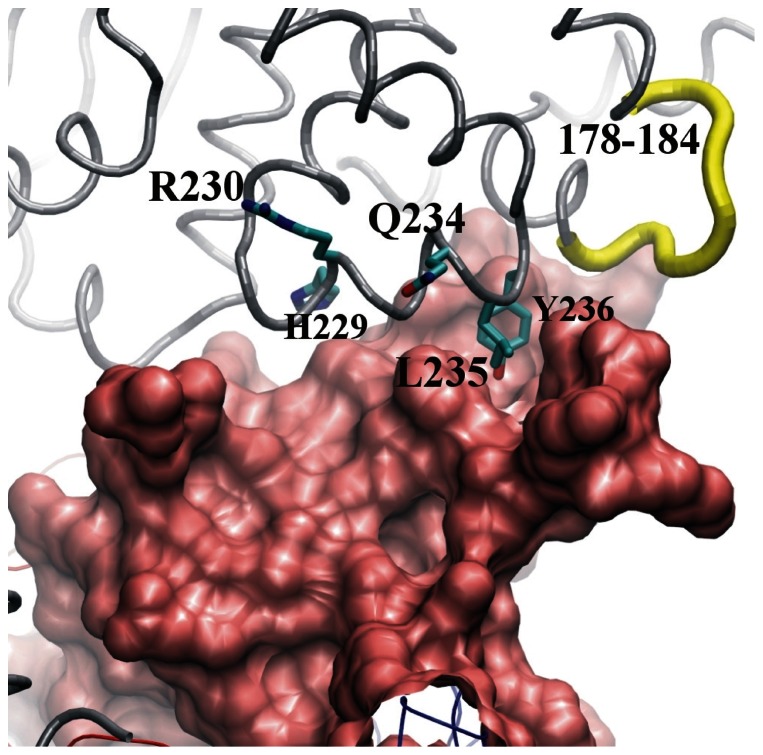
Complex between PA and LF as observed in the X-ray crystal structure 3KWV [**33**] **.** The α carbons constituting LF are drawn as a gray tube, and PA is represented as its molecular surface (pink). The region LF(178–184), the shaving of which abolished reactivity between scFv 2LF and LF, is represented by a yellow tube. Residues of the core of the LF epitope are shown as sticks, colored according to the atom type (carbon in cyan), and are located on α helix 10. The figure was drawn using VMD [50].

Recently a crystal structure consisting of a PA dimer bound to LF_N_ has been resolved, allowing identification of the role of each residue involved in the PA/LF interaction [33]. The hydrophobic residues L188, Y223, H229, V232, L235 and Y236 of LF are closely involved in an interaction with an hydrophobic region of PA. In particular, Y236 appears to be essential by forming an hydrogen-bonding network with PA residues. Thus, targeting three (H229, L235, Y236) of the five LF residues essential for the formation of the lethal toxin (figure 11) is sufficient to destabilize that formation. This mechanism differs from the indirect competition by steric hindrance or conformational changes described as the neutralization mechanisms of other antibodies ([17], also see [12] for a review).

In conclusion, we show in the present study that scFv 2LF cross-neutralizes the edema toxin and the lethal toxin, and we describe a novel *in silico* method based on this cross-reactivity to map its epitopes. This *in silico* method performed better than several existing methods, and may be of more general interest than the present study. The results of the *in silico* method were confirmed and refined by mutations to alanine (alanine shaving followed by alanine scanning), and these methods were found to be more robust than methods based on peptide utilization. The epitopes of scFv 2LF are constituted of LF(229–230) plus LF(234–236) in LF, and of EF(253–254) plus EF(258–260) in EF. These epitopes are located in the region containing helix α10 and it may be possible to target this region with other inhibitors, such as synthetic molecules [34], or by vaccination, to neutralize both LT and ET in order to improve anthrax outcome.

## Materials and Methods

### Ethics Statement

All animal studies presented here were specifically approved by the Institut de Recherche Biomédicale des Armées ethics committee under authorization n° 2006/42.1, and were conducted according to European 2010/63/UE guidelines.

### ELISA and western blot

The cross-reactivity of scFv 2LF with EF was tested by ELISA. Briefly, microtiter plates were coated with 2 µg/ml of EF (List Biological Laboratories, Campbell, CA), while LF (List biological Laboratories) and KLH (Sigma-Aldrich, Saint-Quentin Fallavier, France) were utilized as positive and negative controls respectively. After blocking with PBS-BSA 3%, the plates were incubated with serial dilutions of scFv 2LF. The plates were then washed with PBS-Tween 0.1%, and bound scFv 2LF was detected using a monoclonal anti-polyhistidine antibody conjugated to peroxidase (A7058, Sigma-Aldrich). The reactivity of scFv 2LF with EF was further tested by western blotting under reducing conditions. Three samples of LF and EF were diluted (5, 2 and 1 µg/ml) in RunBlue LDS Sample Buffer (Expedeon, San Diego, USA) with β-mercaptoethanol, heated for 10 min at 80°C and separated by electrophoresis on a 12% SDS-PAGE gel using the Rapid Reducing Running Buffer (Expedeon). The samples were transferred to a polyvinylidene fluoride membrane (Immobilon-Psq, Millipore, Molsheim, France) using a Trans-Blot SD semi-dry electrophoretic transfer cell (Biorad, Marnes-la-Coquette, France). The membrane was blocked, then incubated with a scFv 2LF solution (50 µg/ml). Bound scFv 2LF was revealed with a monoclonal anti-polyhistidine antibody conjugated to peroxidase (A7058, Sigma-Aldrich).

### Affinity measurements

Affinity constants (K_D_) and dissociation constants (k_d_) of scFv 2LF for LF, EF and their variants were measured by surface plasmon resonance (SPR) with a Biacore X (Biacore, Uppsala, Sweden) instrument. ScFv 2LF was immobilized at a maximum of 250 resonance units on a CM5 chip (Biacore) via amine coupling. A flow rate of 30 µl/min was maintained during measurements. A minimum of seven dilutions of LF, EF and their variants (220 to 14 nM) in HBS-EP buffer (Biacore) was tested for 1500 s. After each dilution, the chip was regenerated with glycine 1.5 buffer (Biacore), run at 10 µl/min for 30 s. Results were analyzed with the Biaevaluation software (a 1∶1 (Langmuir) binding model for K_D_ measurements, and a 1∶1 (Langmuir) dissociation model for k_d_ measurements). If an LF or EF variant did not interact with scFv 2LF, its presence was verified by a Biacore experiment using anti-LF polyclonal antibodies. For this, the variant was immobilized on a CM5 chip as described above, and serum drawn from the macaque hyper-immunized with LF was used for detection (diluted 1∶100 in HBS-EP buffer) while the pre-immune serum (diluted 1∶100 in HBS-EP buffer) was used as a control [18].

### *In vivo* neutralization test

ScFv 2LF was reformatted as a full-sized IgG, IgG 2LF, by expression of its variable regions in fusion with human constant regions (Fc) of γ1 and κ isotypes. Human constant regions were preferred to their murine counterparts because the IgG has therapeutic potential, and a human Fc ensures better tolerance. The *in vivo* neutralization test was adapted from a previous study [35]. The edema toxin (ET) was obtained by mixing equal weights of PA and EF. Twentyµg of ET, premixed with 0, 2, 5 or 10 µg of IgG 2LF in a final volume of 25 µl in PBS, or 25 µl of PBS alone as a control, were injected into the right rear footpad of five Balb/c mice. The sizes (dorsal/plantar) of the footpads were measured in triplicate, with digital vernier caliper.

### Identification of epitope candidates by combined identity and solvent exposure analysis

LF and EF sequences were retrieved under the access numbers P15917 and P40136, respectively. The ends of LF_N_ and EF_N_ were defined in accordance with sequence annotations (LF_N_ begins at residue E27 and EF_N_ begins at residue N64) and the two sequences were aligned with the CLUSTALW tool at the PBIL server (http://pbil.univ-lyon1.fr), using standard parameters (figure 4). For each position in LF_N_, a score of 1 or 0 was attributed when the EF_N_ counterpart residue was identical or different, respectively. When alignment gaps occurred, a score of 0 was attributed to the LF residues having no counterparts in EF. A curve averaging the identity percentage over a window of five residues was calculated to visualize the portions shared between EF_N_ and LF_N_, as a length of five residues was considered as corresponding to the length of epitopes [36]. X-ray crystallographic structures for LF (1J7N) [4] and EF (1XFV) [37] were retrieved from the Protein Data Bank (www.rcsb.org). The solvent exposure for each residue on LF_N_ and EF_N_ structures was calculated using the ASAView tool [38], then averaged using a sliding window of five amino acids. The three curves representing these three averaged parameters (identity between LF_N_ and EF_N_, solvent exposure of EF and solvent exposure of LF) are shown in figure 5. Epitope candidates were identified as regions where LF_N_ and EF_N_ identity, averaged on a sliding window of 5 residues, is superior to the mean identity between LF_N_ and EF_N_, and where solvent exposure is high. The identity between LF_N_ and EF_N_, the nature and the solvent exposure of each residue were studied individually to determine the ends of regions selected as epitope candidates. Once the epitope candidates were precisely defined, their solvent exposures were statistically tested (unpaired t test, GraphPad Prism 5, La Jolla, CA).

### Alanine shaving and scanning

Alanine shaving and scanning are two complementary methods, both consisting of directed mutagenesis of residues to alanine to reduce their side chains to methyl groups; this eliminates the interactions involving the side chains of these residues. Alanine shaving is the replacement of several contiguous amino acids in the antigen, and tests for the presence of an epitope in the corresponding region. When alanine shaving indicates that a region contains an epitope, each of the residues has to be individually mutated to alanine to localize the epitope precisely. It might be remarked that mutation to glycine, which has no side-chain beyond the β-carbon, introduces a conformational flexibility which may indirectly alter the epitope and the affinity, so that mutation to glycine is inappropriate for epitope mapping [29].

Six genes, encoding LF and its variants, in which an epitope candidate had been shaved to alanine, were synthesized (Entelechon, Regensburg, Germany), inserted into the vector pET15B (Merck chemical, Darmstadt, Germany) and expressed as described below. The interaction between a variant and scFv 2LF was regarded as significantly altered if the k_d_ was more than a three-fold lower than observed with the parental LF. For alanine scanning, each residue was individually mutated to alanine (Entelechon) and the corresponding variants were expressed as described below. If the mutation of a residue to alanine decreased the k_d_ by more than a three-fold, the mutated residue was regarded as being part of the epitope. All residues identified as composing the epitope were then simultaneously mutated to alanine, and the reactivity of the variant was tested by Biacore. The epitope was confirmed if all immunoreactivity was lost.

Expression and purification of LF, EF and their variants were as described for scFv 2LF [18], except that cytoplasmic extracts were obtained by sonication (1 min pulses of 6 seconds and 10 Watts, separated by pauses of 1 s) (Vibracell 7240S, Bioblock scientifique, Illkirch, France). A band corresponding to each variant was detected by SDS-PAGE in these cytoplasmic extracts, but not in those purified from non-transformed *Escherichia coli* (controls). Purity was estimated at 25% and was insufficient for precise measurement of concentrations, which in turn prevented the measurement of the affinity constant (K_D_). Since the dissociation constant (k_d_) correlates with binding affinity [36] and its measurement does not depend on concentration values, the present study was based on k_d_ measurements.

### Identification of the epitope candidates by existing *in silico* methods

In addition to the *in silico* method developed here, 12 prediction methods available through web servers or stand-alone programs (8 predicting continuous epitopes, 4 predicting discontinuous epitope) were used to identify epitope candidates: BEPITOPE (continuous, Pellequer’s method with TURNEE scale, default settings) [39], BEPITOPE (continuous, Pellequer’s method with TURN33 scale, default settings, [39]), BepiPred (continuous, default settings,[40]), Ellipro (continuous, default settings, [41]), LEP-LP (continuous, default settings, [42]), BCPREDS (continuous, default settings, [43]), Epitopia (continuous, default settings, [44]), COBEpro (continuous, default settings, [45]), CBTOPE (discontinuous, default settings, [46]), Ellipro (discontinuous, default settings, [41]), DiscoTope (discontinuous, default settings, [47]), BEpro (discontinuous, default settings, select residues with score ≥0.5 [48]). A consensus epitope was defined as a region for which half of the methods predicted an epitope.
